# 

**Published:** 1856-04

**Authors:** 


### RECEIPTS ON SUBSCRIPTIONS,

FROM MARCH 15TH TO APRIL 15TH, 1856.

### ILLINOIS.

| | | |
|---|---|---|
| Dr. T. A. Brown, | Vol. 4, | $2 00 |
| " C. C. Allen, | " 5, | 2 00 |
| " Townsend Seely, | " 5, | 2 00 |
| " P. A. Allaire, | Vol. 4 & 5, | 4 00 |
| " J. F. Spelman, | Vol. 4, | 2 00 |
| " J. W. York, | " 5, | 2 00 |
| " M. Wiley, | " 5, | 2 00 |
| " James Leighton, | " 4, | 2 00 |
| " E. J. Tichenor, | " 5, | 2 00 |
| " C. M. Hamilton, | Vol. 4 & 5, | 4 00 |
| " E. E. Webster, | Vol. 4, | 2 00 |
| " W. W. Walker, | " 5, | 2 00 |
| " Ch. Hamnel, | " 5, | 2 00 |
| " Jesse Bennett, | " 5, | 2 00 |
| " A. Hager, | Vol. 4 & 5, | 4 00 |
| " O. S. Jenks, | Vol. 4, | 2 00 |
| " L. Q. Newton, | " 5, | 2 00 |
| " A. Holmes, | " 5, | 2 00 |
| " L. A. Hanneford, | " 5, | 2 00 |
| " G. W. Albin, | " 4, | 2 00 |

### INDIANA.

| | | |
|---|---|---|
| Dr. J. E. Lloyd, | Vol. 4, | $2 00 |
| " F. M. Constant, | " 5, | 2 00 |
| " Jacob Culver, | " 5, | 2 00 |
| " J. Newland, | " 5, | 2 00 |
| " Charles Bracket, | Vol. 3 & 4, | 5 00 |
| " J. R. Robson, | Vol. 5, | 2 00 |

### INDIANA—Continued.

| | | |
|---|---|---|
| Dr. R. D. Mauzy, | " 5, | 2 00 |
| " R. A. Cameron, | " 5, | 2 00 |
| " Jno. Lewis, | " 5, | 2 00 |
| " Geo. Fletcher, | " 4, | 2 00 |
| " J. S. Collings, | " 5, | 2 00 |
| " L. D. Personett, | " 5, | 2 00 |

### WISCONSIN.

| | | |
|---|---|---|
| Dr. A. P. Adams, | Vol. 4, | $2 00 |
| " H. Wearne, | " 5, | 2 00 |
| " John Wiley, | " 5, | 2 00 |

### IOWA.

| | | |
|---|---|---|
| Dr. J. H. Graham, | Vol. 4 & 5, | $4 00 |
| " McCarty, | Vol. 5, | 2 00 |
| " H. Lowe, | " 5, | 2 00 |
| " E. Van Fossen, | " 5, | 2 00 |
| " C. W. Butler, | " 4, | 2 00 |

### MICHIGAN.

| | | |
|---|---|---|
| Dr. J. P. Hall, | Vol. 5, | $2 00 |

### KENTUCKY.

| | | |
|---|---|---|
| Dr. G. M. Huggans, | Vol. 4, | $3 00 |

### CALIFORNIA.

| | | |
|---|---|---|
| Dr. Ira E. Oatman, | Vol. 5, | $2 00 |

